# A Novel *BCR::ABL1* Variant Detected with Multiple Testing Modalities

**DOI:** 10.1155/2024/8486267

**Published:** 2024-08-16

**Authors:** J. Jean, M. Sukhanova, D. Dittmann, J. Gao, L. J. Jennings

**Affiliations:** Department of Pathology Feinberg School of Medicine Northwestern University, Chicago 60611, IL, USA

## Abstract

Chronic myeloid leukemia (CML) is associated with several breakpoint regions that result in different *BCR::ABL1* fusion transcripts. These include the major breakpoint region (M-BCR), minor breakpoint region (m-BCR), and mu breakpoint region (u-BCR) corresponding to p210, p190, and p230 fusion transcripts, respectively. This patient is a 38-year-old female with a new diagnosis of CML in chronic phase. A novel p210 fusion transcript splice variant was detected with qualitative reverse transcription PCR and capillary electrophoresis. Subsequent FISH study was performed, which revealed 86.5% positive for the *BCR::ABL1* fusion. Quantitative real-time polymerase chain reaction (PCR) showed a negative result for the p210 fusion transcript. The variant was further characterized by Sanger sequencing. This variant is in-frame and predicted to be functional. This case illustrates the need for a combination of different testing techniques to fully characterize the rare *BCR::ABL1* fusion transcripts.

## 1. Introduction

CML is a myeloproliferative neoplasm that is most frequently (>95%) diagnosed in the chronic phase. The clinical findings in the chronic phase can range from asymptomatic to splenomegaly, fatigue, malaise, weight loss, night sweats, and anemia [1].

Complete blood counts in CML patients show leukocytosis, absolute basophilia, eosinophilia, and monocytosis. Peripheral blood smears in the chronic phase can demonstrate multiple findings, including increased myelocytes and metamyelocytes and less than 2% blasts [2]. Bone marrow specimens are usually hypercellular, with marked proliferation of granulocytes and expansion at the myelocyte stage. Dysplasia is not significant. Blasts are usually <5% of the marrow cells [3]. However, the morphological findings are nonspecific and diagnosis relies on detection of the *BCR::ABL1* fusion transcript or chromosomal rearrangement.

The *BCR::ABL1* fusion transcript results from the joining of the 5′-end of the *BCR* gene to the 3′-end of the *ABL1* gene. This causes the promoter region of the *BCR* gene to activate the tyrosine kinase domain of the *ABL1* gene. The two most common isoforms of the p210 fusion transcript (M-BCR) are between exon 14 on *BCR* and exon 2 on *ABL1* (e14a2) and between exon 13 on *BCR* and exon 2 of *ABL1* (e13a2). Other isoforms of p210 fusion transcript include e14a3 and e13a3 [4]. The isoforms associated with the p190 fusion transcript (m-BCR) are e1a2 and e1a3. In the p230 fusion transcript (u-BCR), the possible isoforms are e19a2 and e19a3 [1].

According to the European LeukemiaNet organization, a combination of different testing techniques would need to be done to fully characterize the isoforms. These include cytogenetics, interphase FISH, and quantitative real-time and qualitative reverse transcription PCR [5].

## 2. Case Presentation

This is a 38-year-old female with no significant past medical history or family history who presented to the emergency department for lightheadedness for 3 weeks. Peripheral blood smear shows red blood cells with polychromasia and anisopoikilocytosis. Leukocytosis with absolute neutrophilia, monocytosis, and basophilia were noted. A shift to immaturity was seen with the neutrophils, including myelocytes, promyelocytes, and rare blasts suspicious for CML.

## 3. Materials and Methods

**Figure 1:**
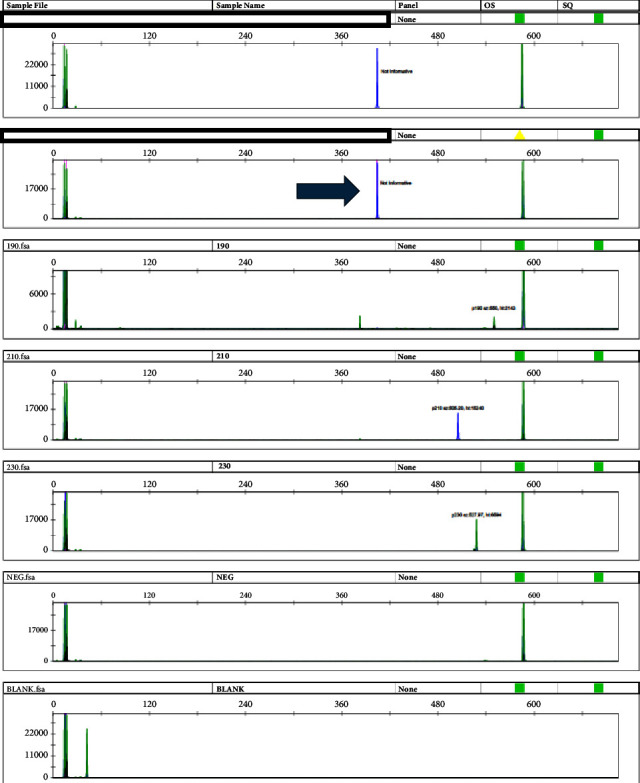
The qualitative reverse transcription PCR shows a positive p210 variant (arrow).

**Figure 2:**
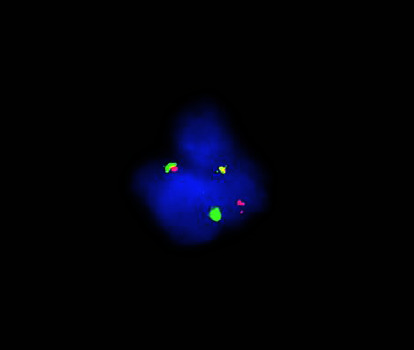
Break-apart FISH test using the BCR probe (green) and the ABL1 probe (orange) reveals a rearrangement with the two genes.

Quantitative PCR was performed on the Cobas z480 analyzer with the Ipsogen BCR-ABL1 Mbcr IS-MMR kit per manufacturer's protocol. Qualitative PCR was performed using long-range reverse transcription PCR, followed by size characterization on the ABI 3130xl Genetic Analyzer using 3 different primer sets with different fluorophores targeting p190, p210, and p230 isoforms (Figure 1). The forward and reverse primer sequences for detecting the p210 fusion transcript on the ABI 3130xl Genetic Analyzer were [FAM] GTGCAGAGTGGAGGGAGAAC and ACTGTTGACTGG-CGTGATGT, respectively. An abnormal reverse transcription PCR product size using the p210 primer set prompted further investigation by Sanger sequencing. Interphase FISH was performed with Vysis probes labeling *ABL1* (9q34) with Spectrum Orange and *BCR* (22q11.2) with Spectrum Green (Figure 2). 200 interphase cells in total were evaluated for each probe by two technologists independently.

## 4. Results

**Figure 3:**
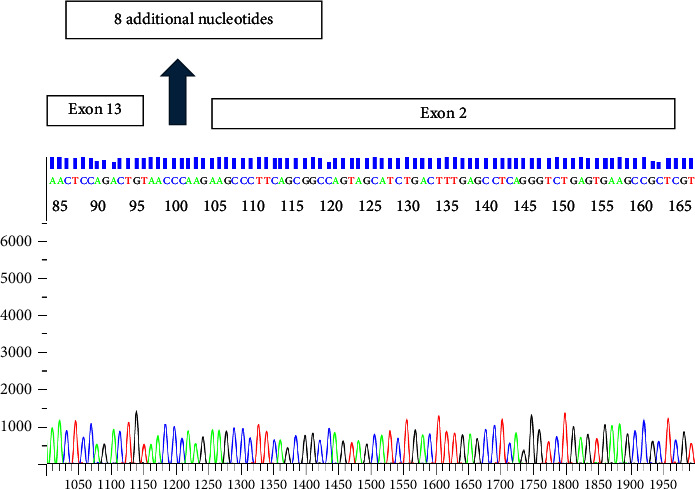
The sequence had been run through BLAST and ENSEMBL software programs. This portion of the electropherogram displays the site between exon 13 from the *BCR* gene and exon 2 of the *ABL1* gene. The eight additional nucleotides–AACCCAAG–detected between the *BCR* and *ABL1* are shown. The sequence AAGCCC is noted to be the first six nucleotides for the *ABL1* gene.

A qualitative reverse transcription PCR test was done, which revealed a positive p210 splice variant. However, the splice variant size did not correspond to the typical isoforms (e14a2, e14a3, e13a2, or e13a3). A subsequent FISH study had confirmed the *BCR::ABL1* rearrangement. The quantitative real-time PCR test used to monitor residual disease and targeting the most common fusion transcript isoforms (e14a2 and e13a2) was negative. Sanger sequencing was done to better characterize the fusion product, which revealed a novel isoform most closely related to the e13a2 isoform. The forward and reverse sequences had been run through a BLAST search, which revealed a missing portion of exon 13. In addition, a novel eight-nucleotide sequence, AACCCAAG, was inserted between the *BCR* and *ABL1* sequences (Figure 3). It had also demonstrated that the forward primer site of the quantitative real-time PCR test was not included in this isoform explaining the negative result.

## 5. Discussion

Variant *BCR::ABL1* transcripts are seen in less than 5% of CML patients, most commonly due to alternative splicing of *BCR* and *ABL1* exons. The most commonly involved isoforms are e6a2, e8a2, e13a3, and e14a3 for p210; e1a2 for p190; and e19a2 for p230 [6]. According to one study by Baccarani et al., the percentage of p190 fusion transcript isoforms is 0.93%, with e1a2 being 0.91% and e1a3 being 0.02%. The percentage of p230 e19a2 isoform is 0.31% [1].

Rare atypical breakpoints have been reported and can be organized into four categories. The first category contains *BCR* breakpoints located within the exons fused to *ABL* a2. The second category is characterized by *BCR* breakpoints located within the introns that are outside of the M-*bcr*, m-*bcr*, or *μ*-*bcr* fused to *ABL* a2. The third category features typical *BCR* breakpoints (M-*bcr*, m-*bcr*, or *μ*-*bcr*) fused to *ABL* breakpoints that are downstream of a2. The fourth category contains transcripts with intervening sequences between *BCR* and *ABL* a2, like the one described herein [7].

This patient had an in-frame fusion transcript that most closely resembles the e13a2 isoform. A partial deletion of exon 13 on *BCR*, insertion of eight additional nucleotides in exon 13 of *BCR*, and unaffected *ABL1* exons 2 and 3 were noted. It has been shown in a previous study that the oncogenic properties of the *BCR::ABL1* fusion product were not affected by the aforementioned changes on exon 13 along with the unaltered *ABL1* exons [8]. In addition, the *ABL1* SH3 domain is theorized to be involved with leukemogenesis by kinase domain (SH1) negative feedback and STAT5 signaling activation [8]. Due to the similarities between the changes seen on exon 13 of *BCR* in this patient and the unaffected *ABL1* gene in both scenarios, it can be speculated that the isoform detected from this patient is functional. The patient subsequently received imatinib, which resulted in a complete cytogenetic and molecular (both p210 and p190) response.

Quantitative PCR has the best analytical measurement range for monitoring CML disease burden [7]. However, the failure to detect less common variants of *BCR::ABL1* in quantitative real-time PCR testing can lead to false negative results. This is due to the PCR primers and probes being limited to their specific target regions. Therefore, according to the European LeukemiaNet organization, it is recommended to simultaneously do additional tests—FISH, cytogenetics, and qualitative PCR—for the diagnosis of CML in the chronic phase [5].

Cytogenetics can detect prognostically significant additional chromosomal aberrations (ACAs) but can miss complex rearrangements resulting in false negative results in up to 5% of the cases. Interphase FISH can detect all of the *BCR::ABL1* rearrangements regardless of the breakpoint region but cannot characterize the isoform [5]. Qualitative reverse transcription PCR can identify and characterize the common and rare *BCR::ABL1* transcript isoforms.

## 6. Conclusion

In summary, we present a patient with CML with a novel *BCR::ABL1* fusion transcript, which is not detectable by quantitative real-time PCR. Diagnosis relied on detection by qualitative reverse transcription PCR and confirmation by FISH. This case highlights the need for a combination of different testing modalities for the diagnosis of CML as recommended by European LeukemiaNet organization.

## Data Availability

The data that support the findings of this study are available from the corresponding author upon reasonable request.
